# Socioeconomic position and its effect on cardiovascular outcomes and mortality in patients with prostate cancer

**DOI:** 10.1093/jncics/pkaf113

**Published:** 2025-11-27

**Authors:** Tarek Nahle, Omar M Makram, Viraj R Shah, Harikrishnan Hyma Kunhiraman, Muhammad Umar Afzal, Irbaz Bin Riaz, Neeraj Agarwal, Umang Swami, Jean-Sebastien Rachoin, Neal L Weintraub, Avirup Guha

**Affiliations:** Division of Cardiology, Department of Medicine, Medical College of Georgia at Augusta University, Augusta, GA, United States; Cardio-Oncology Program, Medical College of Georgia at Augusta University, Augusta, GA, United States; Division of Cardiology, Department of Medicine, Medical College of Georgia at Augusta University, Augusta, GA, United States; Cardio-Oncology Program, Medical College of Georgia at Augusta University, Augusta, GA, United States; Department of Medicine, Medical College of Georgia at Augusta University, Augusta, GA, United States; Division of Cardiology, Department of Medicine, Medical College of Georgia at Augusta University, Augusta, GA, United States; Cardio-Oncology Program, Medical College of Georgia at Augusta University, Augusta, GA, United States; Division of Cardiology, Department of Medicine, Medical College of Georgia at Augusta University, Augusta, GA, United States; Cardio-Oncology Program, Medical College of Georgia at Augusta University, Augusta, GA, United States; Mayo Clinic Comprehensive Cancer Center, Phoenix, AZ, United States; Mayo Clinic Comprehensive Cancer Center, Phoenix, AZ, United States; Division of Medical Oncology, Department of Internal Medicine, Huntsman Cancer Institute, University of Utah, Salt Lake City, UT, Unites States; Division of Medical Oncology, Department of Internal Medicine, Huntsman Cancer Institute, University of Utah, Salt Lake City, UT, Unites States; Department of Medicine, Cooper Medical School of Rowan University, Camden, NJ, United States; Division of Cardiology, Department of Medicine, Medical College of Georgia at Augusta University, Augusta, GA, United States; Cardio-Oncology Program, Medical College of Georgia at Augusta University, Augusta, GA, United States; Division of Cardiology, Department of Medicine, Medical College of Georgia at Augusta University, Augusta, GA, United States; Cardio-Oncology Program, Medical College of Georgia at Augusta University, Augusta, GA, United States

## Abstract

**Background:**

Socioeconomic position (SEP) is a fundamental social determinant of health (SDoH) contributing to observed disparities in cancer and cardiovascular (CV) care among individuals with prostate cancer (PC). Understanding the influence of SEP on CV health is essential for addressing outcome inequities within this population.

**Methods:**

We conducted a retrospective study utilizing Surveillance, Epidemiology, and End Results (SEER)-Medicare to evaluate CV outcomes in patients ≥65 years with PC from 2009 to 2017. We studied the impact of SEP on cardiovascular events (CVEs), cardiovascular mortality (CVm), PC-specific mortality (PCsm), and all-cause mortality. CVE, CVm, and PCsm were analyzed using competing risk models and all-cause mortality with Cox proportional hazards models. A subgroup analysis was performed in Non-Hispanic Black and Non-Hispanic White individuals.

**Results:**

We included 141 242 patients of whom 76 844 were in high SEP areas (45.6%) and 64 398 in low SEP ones (54.4%). Both groups had a mean age of 72 years; patients in low SEP areas were 67.5% Non-Hispanic White, 34.4% having diabetes, 73.3% hypertension, and 67.7% dyslipidemia versus the high SEP group with 85.6% Non-Hispanic White, 30.4% diabetes, 69.9% hypertension, and 70.7% dyslipidemia. Patients in low SEP areas had higher risks of CVm (subdistribution hazard ratio [sHR] = 1.18, 95% CI = 1.12 to 1.25), PCsm (sHR = 1.12, 95% CI = 1.06 to 1.17), CVE (sHR = 1.07, 95% CI = 1.04 to 1.09), and all-cause mortality (HR 1.16, 95% CI = 1.13 to 1.20). Accentuated results were shown in the Non-Hispanic Black subgroup.

**Conclusion:**

Adverse SEP plays a significant role in shaping outcomes among older patients with PC, contributing to elevated risks of adverse CV and survival outcomes.

## Introduction

Prostate cancer (PC) is the most frequently diagnosed cancer among men in the United States, representing a significant public health concern.^1^ Despite advances in treatment and increased survival rates,^2^ mortality in patients with PC is now more commonly attributed to non-cancer causes than to cancer itself.^3^^,^^4^ Notably, cardiovascular disease (CVD) has emerged as the leading cause of death in this population, surpassing PC as the primary contributor to mortality, accounting for up to 30% of all deaths in patients with PC.^4-6^ The surge in CVD in patients suffering from PC can be attributed to shared risk factors such as obesity, age, smoking, genetic predisposition, or to PC itself.^7-9^ Many of the shared factors common to PC and CVD are strongly correlated with social determinants of health (SDoH).^10-12^

While risk factors, genetic predisposition, and treatment-specific adverse events are crucial in the development of CVD,^13-15^ SDoH are emerging as pivotal factors in shaping cardiovascular health among patients with cancer,^16^ and specifically PC.^17-19^ For example, of these SDoH, rurality emerged as an impactful determinant, where patients with PC living in rural areas showed higher risk of CVD.^20^^,^^21^ Similarly, education level interacts with other SDoH and affects cardiovascular outcomes and risks.^21^ Recently, SEP has emerged as a multi-influential SDOH associated with access to care,^22-25^ lifestyle behaviors,^26-28^ exposure to pollution,^29^ and stress levels,^30-33^ all factors known to affect cardiovascular outcomes. Socioeconomic position (SEP), particularly financial resources, plays a critical role in shaping health outcomes. Limited financial means can restrict access to essential healthcare services, nutritious food, and safe living conditions, while increasing exposure to stressors such as job insecurity, poor housing, and unsafe neighborhoods.^34-36^ Over time, these factors contribute to a higher risk of chronic diseases, delayed medical care, and worse overall outcomes.

SEP tends to be more adverse among patients from minority groups, especially Non-Hispanic Black patients, which in turn contributes to healthcare inequity.^37-39^ This disparity is particularly pronounced in PC survival, where disproportionate mortality rates are observed across different racial groups, with the most significant impact seen in the Non-Hispanic Black population,^39-41^ thus adding complexity to the effect of SEP on CVE in Non-Hispanic Black, who are more prone to CVD.^41-44^

Recent conceptual work elaborates the mechanistic pathways through which SEP affects health, specifying interlocking domains of exposure (to risks/resources), susceptibility (biologic vulnerability), social processes (eg, stigma, discrimination), and resilience, operating across individual, interpersonal, and structural levels.^45^ Framed by this model, lower SEP is expected to constrain access to and quality of guideline-concordant care while concurrently heightening stress-inflammatory and metabolic pathways that plausibly exacerbate cardiovascular risk during and after PC therapy.

In this Surveillance, Epidemiology, and End Results (SEER)-Medicare registry study, we aim to understand the impact of SEP on cardiovascular events (CVEs), prostate cancer-specific mortality (PCsm), cardiovascular mortality (CVm), and all-cause mortality in patients with PC. We also aimed to investigate the same association in both Non-Hispanic Black and Non-Hispanic White individuals.

## Methods

### Data source

In this study, we used the SEER-Medicare database and included patients from 2009 through 2017. The SEER-Medicare database includes cases diagnosed from 1999 through 2021, with claims through 2022 and with the last linkage being in 2024. The SEER registry, supported by the National Cancer Institute (NCI), is a comprehensive system of 18 statewide and regional cancer registries across the United States. This network collects and compiles cancer incidence, prevalence, and survival data, representing approximately 47.9% of the US population. SEER captures detailed demographic, clinical, and pathological data, as well as cancer characteristics and survival outcomes for individuals with cancer within their respective registries. The linkage between SEER cancer registries and Medicare claims data creates a comprehensive dataset that pairs clinical cancer information with detailed healthcare utilization, cost, and treatment data for Medicare beneficiaries aged 65 and older.^46^ This integration of both registries constitutes a powerful source of patient baseline characteristics, comorbidities, and outcomes of older patients in the United States.

### Cohort

A PC diagnosis was identified using the International Classification of Diseases for Oncology, Third Edition (ICD-O-3) site recode classification C61. Patients were required to have Medicare Parts A and B and not be members of a health maintenance organization (HMO) for 1 year before and after their PC diagnosis. The identification of comorbidities is not complete for HMO members. In addition, patients should have qualified for Medicare based on age only. Patients were excluded if their cancer was diagnosed at autopsy, their month of cancer diagnosis was missing, if they had a pre-cancerous or in situ lesion only, or had previously been diagnosed with any cancer. Patients with missing follow-up data, neighborhood SEP status, or cancer diagnosis date were excluded from the study. The aim was to study the effect of living in a neighborhood of low SEP on different outcomes in PC patients, including CVE, CVm, PCsm, and all-cause mortality. The follow-up period included PC diagnosis until December 31, 2019, for all studied outcomes. Approval from the institutional board was obtained prior to conducting this study (Institutional IRB ID: 1898238).

### Socioeconomic position

SEP was determined at the census-tract level using the Yost index, which incorporates median house value, median rent, median household income, percent below 150% of poverty line, percent working class, education index, and percent unemployed.^47^ Areas within the first and second quintiles were classified as low SEP, while those in higher quintiles were classified as high SEP.^48^^,^^49^

### Outcomes

The primary outcome of this study was the development at any point during the follow-up period of a CVE, which was defined as atrial fibrillation (AF), heart failure (HF), myocardial infarction (MI), peripheral artery disease (PAD), ischemic stroke (IS), or transient ischemic attack (TIA).^50^^,^^51^ The chronic condition flag file was added to supplement the identification of CVE. Our secondary outcomes were CVm, which included any cause of death related to CVD and defined using ICD-10 code (I00-I99),^52^ in addition to PCsm (defined as primary cause of death being malignant neoplasm of prostate) and all-cause mortality, as displayed in Table S1.

### Covariates

This analysis included various covariates to control for potential confounding factors and ensure a thorough assessment of the relationship between SEP and cardiovascular outcomes. These covariates encompassed demographic factors such as age, race/ethnicity (Non-Hispanic White, Non-Hispanic Black, Hispanic, and other [American Indian, Aleutian, Alaskan Native, Asian Indian, Asian Indian or Pakistani, Chamorro, Chinese, Fiji Islander, Filipino, Guamanian, Hawaiian, Hmong, Japanese, Kampuchean, Korean, Laotian, Micronesian, New Guinean, Other Asian, Pacific Islander, Pakistani, Samoan, Tahitian, Thai, Tongan, Unknown, and Vietnamese]), marital status (single, married including common law, other, and unknown), education level (high education level and low education level), body mass index (BMI), and prior history of CVD.^16^ The category “Other” in the race/ethnicity covariate included non-Hispanic American Indian, Asian, Pacific Islander, or Alaska Native. We also included PC characteristics, which are cancer grade and stage as defined by the SEER combined summary stage. Included relevant comorbidities were diabetes mellitus (DM), hypertension (HTN), hyperlipidemia, and chronic kidney disease (CKD), which were obtained from the Medicare chronic condition files. The patient’s prior PC treatment history was included, comprised of chemotherapy, surgery, radiation therapy, or taking chemical androgen deprivation therapy (ADT) as part of any line of treatment. Education level was based on the Federal Information Processing System (FIPS) Codes for States and Counties and was determined on the level of the county: patients living in counties with high school graduation rates less than 25% were considered to be residing in low education level areas; otherwise, they were considered to be residing in high education counties. Each of these factors was chosen based on its potential to influence both SEP and cardiovascular risk, helping to strengthen the validity of findings regarding the impact of SEP on cardiovascular health. Further descriptions of each covariate are provided in Table S2.

### Statistical analysis

Baseline characteristics for both cohorts of SEP (low and high) were systematically compared. For continuous variables, interquartile ranges (IQR) and median values were used to summarize the central tendency and distribution of the data. The distribution of observations across the categories was presented using frequencies and percentages for categorical variables. Additionally, the Chi-square test was applied to compare categorical variables, while the Mann-Whitney U test was used for continuous variables after concluding a non-normal distribution visually using histograms and statistically using the Shapiro-Wilk test, in order to statistically assess the differences in data distribution across the various categories at baseline. Patients receiving any of the following were considered on ADT: leuprolide acetate, goserelin acetate, abiraterone acetate, flutamide, degarelix acetate, enzalutamide, darolutamide, bicalutamide, and apalutamide. We performed an adjusted competing risk analysis using the Fine and Gray model to obtain the subdistribution hazard ratios (sHRs) for CVE, CVm, and PCsm.^53^ All-cause mortality associated risk was estimated by running Cox proportional hazard models after checking for proportionality assumption and was presented as adjusted hazard ratios (aHR). Our adjustment algorithm included age, race/ethnicity, marital status, education, DM, HTN, hyperlipidemia, CKD, PC grade, PC stage, surgery, chemotherapy, previous radiation treatment, and prior history of CVD.

In order to account for potential effect modification, we conducted interaction testing with several variables that could potentially influence the effect of SEP on our outcomes, and included age over 75 years, race, DM, HTN, PC stage, and rurality. Rurality was similarly assessed at the county level using the US Department of Agriculture Economic Research Service (USDA-ERS)-created Rural-Urban Continuum Codes of 2013 (RUCC), which can identify patients living in rural areas if the RUCC code is greater than or equal to 4.^54^ If the results were statistically significant, stratification was conducted for each tested covariate.

Finally, a sensitivity analysis using a follow-up period of 2 years was conducted for the primary model and interaction testing. Given that most cardiovascular events arise 1–10 years after a PC diagnosis, a window that matches the usual 2-year course of ADT, we performed a sensitivity analysis restricted to the initial 2 years of follow-up to prior to the effect of ADT.^55^

We additionally conducted a subgroup analysis for patients identified as Non-Hispanic Black due to their vulnerability to cardiac disease^56^ and increased PCsm hazard,^57^ as well as Non-Hispanic White individuals.

Analysis was conducted using STATA IC 16.1 (StataCorp, College Station, TX, United States) and the survival and the cmprsk libraries in R 4.4.2,^58^ and we considered a 2-sided *P*-value of <.05 statistically significant. Reporting was done using the STROBE criteria.^59^

## Results

### Population analyzed descriptive statistics

Of all patients in the SEER-Medicare registry, 141 242 patients met our inclusion criteria. The cohort’s median age was 72 years (IQR = 68 to 77), with 77% Non-Hispanic White and 11% Non-Hispanic Black. Patients who were married comprised 48% of all patients, with 54.3% of patients having a high school diploma and 12.4% residing in rural areas. Most patients had local disease (73.6%), followed by regional (10.9%) then distant (8.1%). Patients who underwent surgical intervention were 29.9% of the total, while 33.6% underwent radiation therapy.

Hypertension was the most common associated comorbidity, impacting 71.5% of patients, followed by hyperlipidemia (69.3%), DM (32.2%), and CKD (23.0%) (Table 1).

**Table 1.** Baseline characteristics and cardiovascular outcomes in prostate cancer population stratified by socioeconomic position enrolled in Medicare parts A and B.

Baseline characteristics	Total	High SEP	Low SEP	*P* ^a^
Sample size	141 242	76 844	64 398	
Age at cancer diagnosis, median (IQR)	72 (68-77)	72 (68-76)	72 (68-77)	**<.001**
Race/ethnicity, *n* (%)				**<.001**
Non-Hispanic White	109 298 (77.4%)	65 751 (85.6%)	43 547 (67.6%)	
Non-Hispanic Black	15 474 (11.0%)	4022 (5.2%)	11 452 (17.8%)	
Hispanic	9775 (6.9%)	3314 (4.3%)	6461 (10.0%)	
Other^b^	6695 (4.7%)	3757 (4.9%)	2938 (4.6%)	
Marital status, *n* (%)				**<.001**
Single (Never married)	8432 (6.0%)	3380 (4.4%)	5052 (7.8%)	
Married (including common law)	67 857 (48.0%)	38 736 (50.4%)	29 121 (45.2%)	
Other^c^	13 132 (9.3%)	5793 (7.5%)	7339 (11.4%)	
Unknown	51 821 (36.7%)	28 935 (37.7%)	22 886 (35.5%)	
Education status, *n* (%)				**<.001**
High school and above	76 681 (54.3%)	35 606 (46.3%)	41 075 (63.8%)	
Below high school	64 561 (45.7%)	41 238 (53.7%)	23 323 (36.2%)	
Rurality, *n* (%)				**<.001**
Rural	17 524 (12.4%)	4521 (5.9%)	13 003 (20.2%)	
Urban	123 718 (87.6%)	72 323 (94.1%)	51 395 (79.8%)	
Use of surgery, *n* (%)				**<.001**
No surgery	72 974 (70.1%)	38 157 (69.1%)	34 817 (71.2%)	
Surgery	31 160 (29.9%)	17 043 (30.9%)	14 117 (28.8%)	
Use of radiotherapy, *n* (%)				**<.001**
No radiation/unknown	64 289 (60.9%)	33 563 (60.0%)	30 726 (61.9%)	
Beam radiation	30 709 (29.1%)	16 608 (29.7%)	14 101 (28.4%)	
Implanted radiation	4725 (4.5%)	2595 (4.6%)	2130 (4.3%)	
Other	5859 (5.5%)	3157 (5.6%)	2702 (5.4%)	
ADT, *n* (%)	28 716 (20.3%)	15 017 (19.5%)	13 699 (21.3%)	**<.001**
Chemotherapy, *n* (%)	1166 (1.1%)	631 (1.1%)	535 (1.1%)	**.43**
No treatment, *n* (%)	26 627 (18.9%)	13 643 (24.6%)	12 948 (26.5%)	**<.001**
Staging, *n* (%)				**<.001**
Local	103 979 (73.6%)	56 858 (74.0%)	47 121 (73.2%)	
Regional	15 413 (10.9%)	8857 (11.5%)	6556 (10.2%)	
Distant	11 492 (8.1%)	5592 (7.3%)	5900 (9.2%)	
Unknown	10 358 (7.3%)	5537 (7.2%)	4821 (7.5%)	
Grade, *n* (%)				**<.001**
1	15 289 (10.8%)	8593 (11.2%)	6696 (10.4%)	
2	50 736 (35.9%)	28 244 (36.8%)	22 492 (34.9%)	
3	61 978 (43.9%)	33 436 (43.5%)	28 542 (44.3%)	
4	283 (0.2%)	140 (0.2%)	143 (0.2%)	
N/A	12 956 (9.2%)	6431 (8.4%)	6525 (10.1%)	
Past medical history				
Diabetes, *n* (%)	45 514 (32.2%)	23 335 (30.4%)	22 179 (34.4%)	**<.001**
Hypertension, *n* (%)	100 922 (71.5%)	53 741 (69.9%)	47 181 (73.3%)	**<.001**
Hyperlipidemia, *n* (%)	97 909 (69.3%)	54 307 (70.7%)	43 602 (67.7%)	**<.001**
Chronic kidney disease, *n* (%)	32 459 (23.0%)	15 913 (20.7%)	16 546 (25.7%)	**<.001**
Outcomes				
Any cardiovascular event, *n* (%)				**<.001**
No	75 869 (53.7%)	42 418 (55.2%)	33 451 (51.9%)	
Yes	65 373 (46.3%)	34 426 (44.8%)	30 947 (48.1%)	
Peripheral arterial disease, *n* (%)	17 930 (12.7%)	9018 (11.7%)	8912 (13.8%)	**<.001**
Atrial fibrillation, *n* (%)	31 757 (%)	17 634 (%)	14 123 (%)	**<.001**
Myocardial infarction, *n* (%)	18 266 (12.9%)	9456 (12.3%)	8810 (13.7%)	**<.001**
Ischemic stroke, *n* (%)	16 942 (12.0%)	8710 (11.3%)	8232 (12.8%)	**<.001**
Heart failure, *n* (%)	33 131 (23.5%)	16 632 (21.6%)	16 499 (25.6%)	**<.001**

The bold font represents statistically significant results.

Abbreviations: SEP = socioeconomic position; ADT = androgen deprivation therapy; AJCC = American Joint Committee on Cancer.

a Chi-square test for categorical variables and Mann-Whitney U test for continuous non-normally distributed data.

b Other race/Ethnicity includes: American Indian, Aleutian, Alaskan Native, Asian Indian, Asian Indian or Pakistani, Chamorro, Chinese, Fiji Islander, Filipino, Guamanian, Hawaiian, Hmong, Japanese, Kampuchean, Korean, Laotian, Micronesian, New Guinean, Other Asian, Pacific Islander, Pakistani, Samoan, Tahitian, Thai, Tongan, Unknown, and Vietnamese.

c Other marital status includes separated, divorced, and widowed.

### Patients living in low SEP neighborhoods—descriptive statistics

A total of 76 844 and 64 398 patients resided in high and low SEP neighborhoods, respectively. Those in low SEP neighborhoods were more likely to self-identify as Non-Hispanic Black (17.8% vs. 5.2%, *P* < .001) and Hispanic (10% vs. 4.3%, *P* < .001) and were less likely to be married (45.2% vs. 50.4%, *P* < .001) when compared to patients in high SEP neighborhoods. Additionally, patients residing in low SEP neighborhoods tended to live in rural areas more than their high SEP counterparts (20.2% vs 5.9%, *P* < .001). Patients residing in low SEP neighborhoods were more likely to have DM (34.4% vs. 30.4%, *P* < .001), HTN (73.3% vs. 69.9%, *P* < .001), and CKD (25.7% vs. 20.7%, *P* < .001). PC baseline characteristics as well as management approaches were similar in the 2 SEP groups. A detailed summary of the baseline characteristics of the included patients is provided in Table 1.

### Study outcomes

Throughout the follow-up period, individuals in low SEP neighborhoods tended to have a 7% greater risk of experiencing CVE compared to those residing in high SEP neighborhoods (sHR = 1.07, 95% CI = 1.04 to 1.09). Similarly, they exhibited an 18% greater risk of CVm (sHR = 1.18, 95% CI = 1.12 to 1.25), a 12% greater risk of PCsm (sHR = 1.12, 95% CI = 1.06 to 1.17), and a 16% greater risk of all-cause mortality (HR = 1.13, 95% CI = 1.13 to 1.20) (Table 2).

**Table 2.** Survival analysis using Fine-Gray analysis (competing risk models) and Cox regression for the various cardiovascular outcomes using the fully adjusted model.^a^

	Overall	Non-Hispanic Black	Non-Hispanic White
	**CVE (Competing risk = All-cause mortality) sHR (95% CI,** *P***-value)**		
High SEP	Reference		
Low SEP	**1.07 (1.04 to 1.09, *P* < .001)**	**1.10 (1.02 to 1.19, *P* = .018)**	**1.08 (1.05 to 1.10, *P* < .001)**
	**CVm (Competing risk = All-cause mortality except CVD mortality) sHR (95% CI, *P*-value)**		
High SEP	Reference		
Low SEP	**1.18 (1.12 to 1.25, *P* < .001)**	**1.27 (1.06 to 1.52, *P* = .006)**	**1.17 (1.10 to 1.24, *P* < .001)**
	**PCsm (Competing risk = All-cause mortality except PCsm) sHR (95% CI, *P*-value)**		
High SEP	Reference		
Low SEP	**1.12 (1.06 to 1.17, *P* < .001)**	1.08 (0.92 to 1.26, *P* = .33)	**1.14 (1.08 to 1.19, *P* < .001)**
	**All-cause mortality (Cox) aHR (95% CI, *P*-value)**		
High SEP	Reference		
Low SEP	**1.16 (1.13 to 1.20, *P* < .001)**	**1.17 (1.05 to 1.29, *P* = .003)**	**1.18 (1.14 to 1.22, *P* < .001)**

The bold font represents statistically significant results.

Abbreviations: aHR = adjusted hazard ratio; CVm = cardiovascular mortality; CVE = cardiovascular events; Edu = education; PCsm = prostate cancer-specific mortality; SEP = socioeconomic position; sHR = subdistribution hazard ratio; low education defined as high school education <25%.

a Model adjustment: age, race, marital status, education, prostate cancer grade, prostate cancer stage, diabetes, hypertension, hyperlipidemia, chronic kidney disease, prior history of CVE, surgery, radiation, and chemotherapy use.

For all outcomes, race, rurality, and HTN did not show a significant interaction with living in a low SEP neighborhood. However, a significant interaction was demonstrated with age for CVE, where individuals living in low SEP neighborhoods and below the age of 75 showed a higher risk of CVE (sHR = 1.12, 95% CI = 1.09 to 1.15), unlike those above the age of 75 (sHR = 1.00, 95% CI = 0.97 to 1.04). A similar finding was demonstrated in other outcomes, where those below the age of 75 demonstrated significantly higher CVm (sHR = 1.38, 95% CI = 1.27 to 1.50), PCsm (sHR = 1.29, 95% CI = 1.20 to 1.38), and all-cause mortality (sHR = 1.30, 95% CI = 1.24 to 1.36) compared to those over 75 years. Interaction with DM showed increased risk of CVE only (sHR = 1.10, 95% CI = 1.06 to 1.13), while interaction with PC stage showed increased risk of all-cause mortality for local (sHR = 1.22, 95% CI = 1.17 to 1.26), regional (sHR = 1.17, 95% CI = 1.06 to 1.29), and distant (sHR = 1.07, 95% CI = 1.02 to 1.13) disease (Table 3).

**Table 3.** Interaction testing and consequent stratification for the various cardiovascular outcomes.^a^^,^^b^

	CVE		CVm		PCsm		All-cause mortality	
	sHR (95% CI, *P*-value)						aHR (95% CI, *P*-value)	
Overall Population								
	Interaction	Stratification	Interaction	Stratification	Interaction	Stratification	Interaction	Stratification
Race	*P* = .448	–	*P* = .193	–	*P* = .492	–	*P* = .382	–
Age ≥75	***P* < .001**	**Low SEP + Age <75: 1.12 (1.09 to 1.15, *P* < .001)** Low SEP + Age ≥75: 1.00 (0.97 to 1.04, *P* = .72)	***P* < .001**	**Low SEP + Age <75: 1.38 (1.27 to 1.50, *P* < .001)** **Low SEP + Age ≥75: 1.07 (1.01 to 1.15, *P* = .026)**	***P* < .001**	**Low SEP + Age <75: 1.29 (1.20 to 1.38, *P* < .001)** Low SEP + Age ≥75: 1.03 (0.97 to 1.10, *P* = .41)	***P* < .001**	**Low SEP + Age <75: 1.30 (1.24 to 1.36, *P* < .001)** **Low SEP + Age ≥75: 1.09 (1.05 to 1.13, *P* < .001)**
Rurality	*P* = .86	–	*P* = .87	–	*P* = .26	–	*P* = .20	–
DM	***P* = .037**	**Low SEP + No DM: 1.05 (1.02 to 1.08, *P* < .001)** **Low SEP + DM: 1.10 (1.06 to 1.13, *P* < .001)**	*P* = .98	–	*P* = .33	–	*P* = .09	–
HTN	*P* = .43	–	*P* = .20	–	*P* = .88	–	*P* = .16	–
PC Stage	*P* = .15	–	*P* = .18	–	*P* = .062	–	***P* < .001**	**Low SEP + stage localized: 1.22 (1.17 to 1.26, *P* < .001)** **Low SEP + stage regional: 1.17 (1.06 to 1.29, *P* = .002)** **Low SEP + stage distant: 1.07 (1.02 to 1.13, *P* = .010)**
Non-Hispanic Blacks								
	Interaction	Stratification	Interaction	Stratification	Interaction	Stratification	Interaction	Stratification
Age ≥75	***P* = .045**	**Low SEP + Age <75: 1.16 (1.06 to 1.27, *P* = .002)** Low SEP + Age ≥75: 0.98 (0.86 to 1.12, *P* = .816)	*P* = .062	–	***P* = .014**	**Low SEP + Age <75: 1.33 (1.07 to 1.67, *P* = .012)** Low SEP + Age ≥75: 0.91 (0.73 to 1.13, *P* = .243)	*P* = .038	**Low SEP + Age <75: 1.28 (1.12 to 1.47, *P* < .001)** Low SEP + Age ≥75: 1.03 (0.89 to 1.20, *P* = .841)
Rurality	*P* = .35	–	*P* = .64	–	*P* = .804	–	*P* = .32	–
DM	*P* = .092	–	*P* = .52	–	*P* = .009	**Low SEP + No DM: 1.26 (1.03 to 1.54, *P* = .023)** Low SEP + DM: 0.84 (0.66 to 1.06, *P* = .144)	*P* = .17	–
HTN	*P* = .46	–	*P* = .44	–	*P* = .58	–	*P* = .35	–
PC Stage	*P* = .71	–	*P* = .57	–	*P* = .37	–	***P* = .0435**	**Low SEP + stage localized: 1.26 (1.11 to 1.44, *P* = .001)** Low SEP + stage regional: 1.50 (0.96 to 2.33, *P* = .002) Low SEP + stage distant: 1.00 (0.81 to 1.25, *P* = .967)
Non-Hispanic White								
	Interaction	Stratification	Interaction	Stratification	Interaction	Stratification	Interaction	Stratification
Age ≥75	***P* < .001**	**Low SEP + Age <75: 1.13 (1.09 to 1.16, *P* < .001)** Low SEP + Age ≥75: 1.01 (0.98 to 1.05, *P* = .436)	***P* = .003**	**Low SEP + Age <75: 1.31 (1.19 to 1.45, *P* < .001)** **Low SEP + Age ≥75: 1.09 (1.02 to 1.17, *P* = .012)**	***P* < .001**	**Low SEP + Age <75: 1.31 (1.22 to 1.42, *P* < .001)** Low SEP + Age ≥75: 1.05 (0.99 to 1.11, *P* = .086)	***P* < .001**	**Low SEP + Age <75: 1.29 (1.23 to 1.36, *P* < .001)** **Low SEP + Age ≥75: 1.12 (1.08 to 1.16, *P* < .001)**
Rurality	*P* = .601	–	*P* = .743	–	*P* = .119	–	*P* = .444	–
DM	*P* = .583	–	*P* = .908	–	*P* = .636	–	*P* = .597	–
HTN	*P* = .749	–	*P* = .649	–	*P* = .922	–	*P* = .368	–
PC Stage	***P* = .002**	**Low SEP + Local: 1.09 (1.06 to 1.11, *P* < .001)** **Low SEP + Regional: 1.15 (1.07 to 1.23, *P* < .001)** Low SEP + Distant: 0.96 (0.90 to 1.04, *P* = .317)	***P* = .034**	**Low SEP + Local: 1.22 (1.14 to 1.31, *P* < .001)** Low SEP + Regional: 1.20 (0.96 to 1.50, *P* = .111) Low SEP + Distant: 0.99 (0.83 to 1.18, *P* = .937)	*P* = .195	–	***P* = .017**	**Low SEP + Local: 1.23 (1.18 to 1.28, *P* < .001)** **Low SEP + Regional: 1.19 (1.06 to 1.33, *P* = .003)** **Low SEP + Distant: 1.10 (1.03 to 1.17, *P* = .002)**

The bold font represents statistically significant results. Further stratification was only done if interaction was found to be significant.

Abbreviations: aHR = adjusted hazard ratio; CVm = cardiovascular mortality; CVE = cardiovascular events; DM = diabetes mellitus; HTN = hypertension; PC = prostate cancer; PCsm = prostate cancer-specific mortality; sHR = subdistribution hazard ratio.

a Model adjustment: age, race, marital status, education, prostate cancer grade, prostate cancer stage, diabetes, hypertension, hyperlipidemia, chronic kidney disease, prior history of CVE, surgery, radiation, and chemotherapy use.

b In case of no significance for interaction analysis, stratification was not conducted.

Similar findings were observed after the sensitivity analysis with a 2-year follow-up period for the main population (Table S3). Interaction testing for sensitivity analysis showed similar results for the overall population, with HTN gaining significance for all-cause mortality, and DM losing its significance (Table S4).

### Study outcomes—Non-Hispanic Black subgroup

In the Non-Hispanic Black subgroup, individuals living in low SEP neighborhoods showed an increased risk of CVE of 10% (sHR = 1.10, 95% CI = 1.02 to 1.19). Similarly, there was an increased risk of CVm by 27% (sHR = 1.27, 95% CI = 1.06 to 1.52), and all-cause mortality of 17% (sHR = 1.17, 95% CI = 1.05 to 1.29) with no significant increase in PCsm risk (Table 2).

Rurality and HTN did not show any interaction with SEP for any of the outcomes. Age below 75, however, significantly interacted with low SEP for CVE (sHR = 1.16, 95% CI = 1.06 to 1.27), PCsm (sHR = 1.33, 95% CI = 1.07 to 1.67), and all-cause mortality (sHR = 1.28, 95% CI = 1.12 to 1.47). Having no DM increased the risk of PCsm for those living in low SEP neighborhoods (sHR = 1.26, 95% CI = 1.03 to 1.54). For PC stage, only localized stage showed an interaction (sHR = 1.26, 95% CI = 1.11 to 1.44) (Table 3).

Our sensitivity analysis aiming to evaluate robustness and consistency of our observations in the Non-Hispanic Black subgroup showed loss of significance for CVE, while maintaining significance for other outcomes and gaining significance for PCsm (Table S3). Additionally, sensitivity analysis for interaction with age showed loss of significance for CVE and PCsm while gaining significance for CVm (sHR = 1.72, 95% CI = 1.19 to 2.48) (Table S4).

### Study outcomes—Non-Hispanic White subgroup

In the Non-Hispanic White subgroup, an increased risk of 8% was seen for CVE (sHR = 1.08, 95% CI = 1.0 to 1.10). Similarly, there was an increase in CVm risk of 17% (sHR = 1.17, 95% CI = 1.10 to 1.24), PCsm of 14% (sHR = 1.14, 95% CI = 1.08 to 1.19), and all-cause mortality of 18% (sHR = 1.18, 95% CI = 1.14 to 1.22) (Table 2).

Rurality, DM, and HTN did not show any interaction with SEP for any of the outcomes. Age below 75 years, however, was shown to interact with SEP for CVE (sHR = 1.13, 95% CI = 1.09 to 1.16), CVm (sHR = 1.31, 95% CI = 1.19 to 1.45), PCsm (sHR = 1.31, 95% CI = 1.22 to 1.42), and all-cause mortality (sHR = 1.29, 95% CI = 1.23 to 1.36). Similarly, PC stage showed a positive interaction in earlier stages in CVE, CVm, and all-cause mortality (Table 3). Sensitivity analysis in this population showed loss of significance for PC stage interaction in CVm and all-cause mortality (Table S4).

## Discussion

Our study was the first to assess the impact of SEP on different outcomes in patients with PC, and the subgroup of patients identifying as Non-Hispanic Black, while adjusting for relevant covariates. Although previous studies assessed cardiac mortality in men with PC, our study was the first one to explore cardiac and cancer specific outcomes in a competing approach, with subgroup analyses and interactions with other factors including comorbidities and cancer characteristics, as well as a time-based sensitivity analysis. Our analysis revealed an increased risk of CVE, CVm, PCsm, and all-cause mortality in patients with PC living in low SEP neighborhoods. We also demonstrated an increase in the risk of CVE, CVm, and all-cause mortality that was more pronounced in patients identifying as Non-Hispanic Black, while having similar results between Non-Hispanic White and the overall population, due to them constituting 77% of the studied cohort. Our findings showcase the importance of considering financial means in individuals as well as their living conditions and environment as crucial clinical factors which could impact morbidity and mortality in patients with PC. These findings could imply a different approach in treatment and management of these patients, especially in populations who are more vulnerable, such as Non-Hispanic Black.^60^

In addition to our main findings, our study showed a positive interaction between living in a low SEP area and age <75, which was associated with increased risk of all outcomes. Also, we found that low SEP interacted with DM to increase CVE, and with PC stage to increase the risk of all-cause mortality across all stages, more so in earlier stages than later ones. In the Non-Hispanic Black subgroup, low SEP interacted with age <75 to increase CVE, PCsm, and all-cause mortality, while no DM increased PCsm. Adding a short term (2-year follow-up only) sensitivity analysis showed a loss of significance for DM in the overall population and a gain of significance of HTN for all-cause mortality. For Non-Hispanic Black, sensitivity analysis for interaction only showed age <75 being significant for CVm.

In prior studies, low SEP has been linked to increased CVD in men and women.^61^ Higher income countries also show an inverse relationship between socioeconomic status, cardiovascular risk factors (CVRFs), and CVD.^62^ This study found that the influence of SEP on CVD in the general population also applies to patients suffering from PC. Other studies explicitly addressed socioeconomic status’s influence on outcomes of patients with PC. For instance, Tombic et al.^63^ observed that in Sweden, patients with a higher socioeconomic status had a lower PCsm and all-cause mortality, lower waiting times, and lower risk of positive margins. Screening is similarly affected, where men with higher incomes are more likely to participate in screening than those with lower incomes, leading to a lower incidence of high-risk PC.^64^ Risk factors such as obesity are also enhanced by poverty, which makes them harder to control. As these are shared risk factors between CVD and PC, living in low SEP areas is associated with increased frequency and severity of both.^65^

In the sensitivity analysis, it was shown that the increased risk of CVE lost its significance in the Non-Hispanic Black subgroup, which could be explained by a divergence in rates of incidence over time, as CVEs tend to develop over long durations. Another potential explanation could be loss of power due to the small number of Non-Hispanic Black patients.

Furthermore, this increased risk is accentuated by being younger than 75 years of age, where CVE, CVm, PCsm, and all-cause mortality increase significantly for these younger patients when compared to patients older than 75 years in low SEP neighborhoods. This might be explained by a multitude of factors, including the natural progression of age-related physiological changes, such as vascular aging or reduced metabolic reserve, which might overshadow the incremental impact of socioeconomic disparities on cardiovascular risk.^66^ Alternatively, differences in levels of stress, access to care, etc might contribute to the interaction between age and cardiovascular health in men with PC.^67^^,^^68^ Additionally, CVm and all-cause mortality increase as well in patients older than 75 years but to a lesser extent, suggesting that older men may be less affected by their SEP. We also showed an effect of DM, which worsened cardiovascular morbidity by increasing the risk of CVE. This was not shown in the 2 year sensitivity analysis, possibly due to length of time required for DM to promote CVD.^69^ Regarding PC stage, earlier stages showed increased all-cause mortality compared to later stages. Later stages carry a worse prognosis that likely outweighs the adverse effects of low socioeconomic status, and thus diminishes the latter’s impact on mortality.

In the Non-Hispanic Black subgroup, not having DM increased the risk of PCsm. Vulnerable groups such as Non-Hispanic Black have less access to healthcare, and those with DM are typically followed-up more frequently, leading to increased interaction with medical facilities. Due to the closer and more accessible follow-up of DM patients as described by Paddison et al,^70^ PC screening, referral, and management may be enhanced in this subgroup.

This study highlights the dynamics between different SDoH and their effect on overall health. Historical neighborhood redlining may reduce access to care and was shown to increase CV risk factors and cardiometabolic disease.^71^ SDoH play a major role in affecting CVRFs and outcomes; for example, the Protocol for Responding to & Assessing Patients’ Assets, Risks & Experiences (PRAPARE) questionnaire, which integrates multiple SDoH, was shown to predict uncontrolled HTN in patients with cancer.^43^ Furthermore, race itself is related closely with income, which impacts SEP^72^ and other SDoH.^73^ An adequate course of action would be the implementation of and electronic health record triggered cardio-oncology care pathway for men with lower SEP (ICD-10 Z-codes based), which would increase the cardiac specific screening and care in vulnerable patients.^74^

Although higher SEP may have a protective effect in Non-Hispanic Black men, the limits of protection should be acknowledged due to the persistence of additional structural barriers and systemic biases for this population. A holistic approach would be appropriate to target these barriers, from proper patient education, facilitation of access to care, and targeting other SDoH.

This study has several limitations. SEP and education level data were obtained at the census tract/county level due to the unavailability of individual-level data points, in addition to the unidimensional measurement of SEP, which should be measured using multiple components such as Area Deprivation Index and NCI census block indicators in further studies. Second, SEER-Medicare includes only patients aged 66 years and older, which leaves patients less than 66 years of age not represented in our study. Third, this study is observational; thus, no direct causal relationship can be established, only associations. Fourth, while combining SEER registry data with Medicare information can broaden the extent of available data, integrating 2 distinct sources may introduce potential misjudgments or inconsistencies. Fifth, because of the retrospective design of SEER-Medicare, certain variables of interest were not available, and some information may be incomplete or missing, such as information regarding radiation therapy and chemotherapy, with concern regarding completeness of the variables and biases associated with receiving or not receiving treatment. Additionally, unavailability of PC specific data such as prostate specific antigen data or Gleason scores rendered us unable to adequately stratify patients. Sixth, excluding patients with missing follow-up data might cause selection bias, on top of using ICD codes which may lead to missing some patients. Seventh, although a wide range of covariates were included to account for potential confounders and enhance the validity of our findings, the possibility of residual confounding cannot be excluded. Unmeasured or inadequately measured variables may still influence the observed associations. However, STROBE guidelines were followed to mitigate biases noted in observational studies. Finally, SEP is a long-term exposure that interacts with other SDoH. Future studies should explore the interplay of SDoH in CVD development among PC patients.

## Conclusion

Our study demonstrates a significant association between living in low SEP neighborhoods and adverse outcomes in patients with PC, including increased risks of CVE and mortality. These associations are particularly pronounced in the Non-Hispanic Black subgroup, highlighting the compound effect of socioeconomic disparities on vulnerable PC populations. These findings strongly suggest the need for targeted medical and social interventions and enhanced cardiovascular surveillance in PC patients from lower socioeconomic backgrounds. The findings further call for policy initiatives to address healthcare access barriers to improve CV outcomes in PC patients.

## Supplementary Material

pkaf113_Supplementary_Data

## Data Availability

Access to data is available upon request through the SEER-Medicare website, researchers are required to complete an application process with the National Cancer Institute to request the data (https://healthcaredelivery.cancer.gov/seermedicare/overview/).
