# Supplementary material for: Socioeconomic position and its effect on cardiovascular outcomes and mortality in patients with prostate cancer
Source: JNCI Cancer Spectr. 2025 Nov 27;9(6):pkaf113. doi: 10.1093/jncics/pkaf113 (PMC12729912; doi:10.1093/jncics/pkaf113)
Supplement: pkaf113_Supplementary_Data [file pkaf113_supplementary_data.pdf]

Supplementary Tables

Table S1. Description of exposure and outcomes, and their definitions.

Variables	Explanation and ICD-9 and ICD-10 codes used
Exposure	
Socioeconomic Yost Index	Measuring socioeconomic status across geographic areas.
Outcomes	
Heart failure	428.XX, 402.01, 402.11, 402.91, 414.8, I50.XX, I11.00, I13.0, I13.2, I42.0, I42.5, I42.6, I42.7, I42.8, I42.9, I43.XX
Ischemic stroke	431.XX, 435.0X, 435.1X, 435.2X, 435.3X, 435.8X, 435.9X, 433.01, 433.11, 433.21, 433.31, 433.81, 433.91, 434.01, 434.11, 434.91, 997.01, 344.60, 344.61, I619, G45X, I63, I66, G834, G9781
Acute myocardial infarction	411.XX, 410.XX, I20.0, I21.X, I24.X
Atrial fibrillation	427.31, I48.91
Peripheral artery disease	440.2×, 440.3×, 440.8×, 440.9×, 443.9×, I70.0; I70.2; I73.9
CVm	I00-I09, I11, I13, I20-I51, I60-169
PCsm	C61

ICD: International classification of diseases; CVm: Cardiovascular mortality; PCsm: Prostate Cancer Specific Mortality

Table S2. Description of the different covariates and their definitions.

Variables	Explanation
Covariates	
Age at diagnosis	Study includes only patients aged 65 years and above.
Race	Recoded field with classification as:<br>• White (reference)<br>• Black<br>• Other
Ethnicity	Hispanic; non-Hispanic
Marital status	Marital Status has the following categories:<br>• Unmarried-Single (reference)<br>• Married<br>• Other<br>• Unknown
Rurality	Rurality status (yes vs no) was defined according to 2013 Rural-Urban Continuum Codes (RUCC).<br><br>It is a categorization system that differentiates metropolitan counties based on the population size of their metropolitan area and classifies nonmetropolitan counties according to their level of urbanization and proximity to a metropolitan area<br><br>Positive rural status: patients residing in areas with a population of <2,500 or <20,000 not adjacent to metropolitan area (according to RUCC codes 7,8, or 9)

County level educational status	Percentage of people >25 years old with less than a high school diploma and percentage of people >25 years old with only a high school diploma
Chronic Kidney Disease	Classified as:<br>• 0 (reference; no history of diagnosis or diagnosis after prostate cancer diagnosis)<br>1 (history of diagnosis before the prostate cancer diagnosis)
Hypertension	Classified as:<br>• 0 (reference; no history of diagnosis or diagnosis after prostate cancer diagnosis)<br>• 1 (history of diagnosis before the prostate cancer diagnosis)
Diabetes mellitus	ICD Codes 250.xx and 362.0x and chronic condition flag files
Hyperlipidemia	Classified as:<br>• 0 (reference; no history of diagnosis or diagnosis after prostate cancer diagnosis)<br>• 1 (history of diagnosis before the prostate cancer diagnosis)
SEER combined summary stage	Combination of NAACCR Items #759 and #3020. For 2004-2015 cases, it is copied from Derived SS2000 and for 2016+, it is the best available between Derived SS2000 and SEER Summary Stage 2000. All benign/borderline cases are set to NA<br>• Local 0,1(reference; 0 = In Situ, 1= Localized at the time of diagnosis)<br>• Regional 2,3,4,5 (2 Regional- direct extension only, 3 Regional- regional lymph nodes only, 4 Regional- direct extension and regional nodes only, 5 Regional- NOS)<br>• Distant 7 · Unknown 8,9 (8 Not applicable 9 Unknown/Unstaged)
Histology	

Tumor Grade	Grade at prostate cancer diagnosis categorized as:<br>• 1 (reference)<br>• 2<br>• 3<br>• 4
Androgen Deprivation Therapy	Classified as:<br>• 0 (did not receive leuprolide ADT)<br>• 1 (received leuprolide ADT)
Specific Androgen Deprivation Therapy Medications	Apalutaminde, Bicalutamide, Darolutamide, Degarelix acetate, Enzalutamide, Flutamide, Goserelin acetate, Leuprolide acetate
Chemotherapy	Classified as:<br>• 0 (did not receive chemotherapy)<br>• 1 (received chemotherapy)
Radiotherapy	Classified as:<br>• 0 (did not receive radiotherapy)<br>• Beam radiation<br>• Implanted radiation
Surgery	Classified as:<br>• 0 (reference)<br>• Radical prostatectomy<br>• Laparoscopic prostatectomy<br>• Bilateral orchiectomy<br>• TURP

ADT: Androgen deprivation therapy; CVD: cardiovascular disease; TURP: transurethral resection of the prostate.

Table S3. Sensitivity analysis using Fine-Gray analysis (competing risk models) and Cox regression for the various cardiovascular outcomes using the fully adjusted model⁺ within two years of start of follow-up. The bold font represents statistically significant results.

	Overall	NHB	NHW
	**CVE (Competing risk = All-cause mortality) sHR (95% CI, p-value)**		
High SEP	Reference		
Low SEP	**1.07 (1.03-1.10, p<0.001)**	1.06 (0.94-1.19, p=0.32)	**1.08 (1.05-1.10, p<0.001)**
	**CVm (Competing risk = All-cause mortality except CVD mortality) sHR (95% CI, p-value)**		
High SEP	Reference		
Low SEP	**1.17 (1.08-1.25, p<0.001)**	**1.32 (1.03-1.70, p=0.03)**	**1.17 (1.10-1.24, p<0.001)**
	**PCsm (Competing risk = All-cause mortality except PCsm) sHR (95% CI, p-value)**		
High SEP	Reference		
Low SEP	**1.16 (1.09-1.22, p<0.001)**	**1.25 (1.02-1.53, p=0.031)**	**1.14 (1.08-1.19, p<0.001)**
	**All-cause mortality (Cox) aHR (95% CI, p-value)**		
High SEP	Reference		
Low SEP	**1.16 (1.11-1.22, p<0.001)**	**1.28 (1.09-1.50, p=0.003)**	**1.18 (1.12-1.24, p<0.001)**

aHR: adjusted hazard ratio; CVm: cardiovascular mortality; CVE: cardiovascular events; Edu: education; NHB: non-Hispanic Blacks; PCsm: prostate cancer-specific mortality; SEP: socioeconomic position; sHR: subdistribution hazard ratio; Low Education defined as high school education <25%.

⁺Model adjustment: Age, race, marital status, education, prostate cancer grade, prostate

cancer stage, diabetes, hypertension, hyperlipidemia, chronic kidney disease, prior history of CVE, surgery, radiation, and chemotherapy use.

Table S4. Interaction testing and consequent stratification for the various cardiovascular outcomes for the sensitivity analysis (within two years of follow-up)[+]. The bold font represents statistically significant results. Further stratification was only done if interaction was found to be significant.

	CVE		CVm		PCsm		All-cause Mortality	
	**sHR (95% CI, p-value)**						**aHR (95% CI, p-value)**	
Overall Population								
	Interaction	Stratification	Interaction	Stratification	Interaction	Stratification	Interaction	Stratification
Race	P=0.722	-	P=0.489	-	P=0.544	-	P=0.819	-
Age ≥75	**P<0.001**	**Low SEP + Age <75: 1.13 (1.09-1.18, P<0.001)**<br>Low SEP + Age ≥75: 1.01 (0.96-1.05, P=0.82)	**P=0.002**	**Low SEP + Age <75: 1.36 (1.21-1.53, P<0.001)**<br>Low SEP + Age ≥75: 1.08 (0.99-1.18, P=0.09)	**P<0.001**	**Low SEP + Age <75: 1.34 (1.22-1.48, P<0.001)**<br>**Low SEP + Age ≥75: 1.08 (1.01-1.16,**	**P<0.001**	**Low SEP + Age <75: 1.34 (1.24-1.45, P<0.001)**<br>**Low SEP + Age ≥75: 1.09 (1.04-1.15,**

						**P=0.028)**		**P=0.001)**
Rurality	P=0.78	-	P=0.30	-	P=0.53	-	P=0.27	-
DM	P=0.72	-	P=0.89	-	P=0.38	-	P=0.09	-
HTN	P=0.68	-	P=0.051	-	P=0.63	-	**P=0.019**	**Low SEP + No HTN: 1.31 (1.17-1.46, P<0.001)**<br>**Low SEP + HTN: 1.13 (1.08-1.19, P<0.001)**
PC Stage	P=0.14	-	P=0.84	-	P=0.14	-	**P=0.025**	**Low SEP + stage localized: 1.22 (1.13-1.31, P<0.001)**<br>**Low SEP + stage regional: 1.34 (1.12-1.61,**

								**P=0.001)** **Low SEP + stage distant: 1.10 (1.03-1.17, P=0.006)**
Non-Hispanic Blacks								
	Interaction	Stratification	Interaction	Stratification	Interaction	Stratification	Interaction	Stratification
Age ≥75	P=0.079	-	**P=0.044**	**Low SEP + Age <75: 1.72 (1.19-2.48, P=0.004)** Low SEP + Age ≥75: 1.02 (0.73-1.44, P=0.898)	P=0.14	-	P=0.059	**-**
Rurality	P=0.88	-	P=0.91	-	P=0.63	-	P=0.26	-
DM	P=0.75	-	P=0.19	-	P=0.064	**-**	P=0.074	-
HTN	P=0.39	-	P=0.84	-	P=0.35	-	P=0.18	-

PC Stage	P=0.81	-	P=0.65	-	P=0.078	-	P=0.061	-
Non-Hispanic Whites								
	Interaction	Stratification	Interaction	Stratification	Interaction	Stratification	Interaction	Stratification
Age ≥75	**P<0.001**	**Low SEP + Age <75: 1.13 (1.09-1.16, P<0.001)** Low SEP + Age ≥75: 1.01 (0.98-1.05, P=0.436)	**P=0.003**	**Low SEP + Age <75: 1.31 (1.19-1.45, P<0.001) Low SEP + Age ≥75: 1.09 (1.02-1.17, P=0.012)**	**P=0.003**	**Low SEP + Age <75: 1.31 (1.22-1.42, P<0.001)** Low SEP + Age ≥75: 1.05 (0.99-1.11, P=0.086)	**P=0.007**	**Low SEP + Age <75: 1.31 (1.19-1.43, P<0.001) Low SEP + Age ≥75: 1.13 (1.06-1.20, P<0.001)**
Rurality	P=0.601	-	P=0.743	-	P=0.119	-	P=0.299	-
DM	P=0.583	-	P=0.908	-	P=0.636	-	P=0.814	-
HTN	P=0.749	-	P=0.649	-	P=0.922	-	P=0.095	-
PC Stage	P=0.002	**Low SEP + Local: 1.08 (1.06-1.11, P<0.001) Low SEP + Regional: 1.15 (1.07-1.23, P<0.001)** Low SEP + Distant: 0.96 (0.90-1.04, P=0.326)	P=0.108	-	P=0.195	-	P=0.339	-

aHR: adjusted hazard ratio; CVm: cardiovascular mortality; CVE: cardiovascular events; DM: diabetes mellitus; HTN: hypertension; PC: prostate cancer; PCsm: prostate cancer-specific mortality; sHR: subdistribution hazard ratio

[+]Model adjustment: Age, race, marital status, education, prostate cancer grade, prostate cancer stage, diabetes, hypertension, hyperlipidemia, chronic kidney disease, prior history of CVE, surgery, radiation, and chemotherapy use.
